# Evaluation of Three Recombinant Antigens for the Detection of Anti-Coxiella Antibodies in Cattle

**DOI:** 10.3390/antib14040107

**Published:** 2025-12-12

**Authors:** Barbara Colitti, Consiglia Longobardi, Gabriela Flores-Ramirez, Chiara Nogarol, Ludovit Skultety, Gianmarco Ferrara

**Affiliations:** 1 Department of Veterinary Science, University of Turin, 10095 Grugliasco, Italy; barbara.colitti@unito.it; 2 Department of Veterinary Medicine and Animal Productions, University of Naples, “Federico II”, 80138 Naples, Italy; consiglia.longobardi@unina.it; 3 Institute of Virology, Slovak Academy of Sciences, 84505 Bratislava, Slovakia; virugafl@savba.sk (G.F.-R.);; 4 Specialist Virology Unit, Istituto Zooprofilattico Sperimentale del Piemonte, Liguria e Valle d’Aosta (IZSPLV), Via Bologna 148, 10154 Torino, Italy; chiara.nogarol@izsplv.it; 5 Department of Veterinary Sciences, University of Messina, 98122 Messina, Italy

**Keywords:** *Coxiella burnetii*, recombinant antigen, ELISA, antibodies, Q fever, AdaA, Com-1, MceB

## Abstract

**Background/Objectives:** The detection of anti-Coxiella antibodies using serological methods is essential for identifying exposed ruminants and preventing this important zoonotic disease in livestock. In recent years, numerous attempts have been made to increase diagnostic performance as well as simplify the production of serological assays. Commercially available tests often use whole-cell antigens, which can decrease specificity and require high-level biosafety facilities for manufacturing. The aim of this work was to produce three *Coxiella burnetii* (*C. burnetii*) antigens in recombinant form and assess them for the detection of anti-Coxiella antibodies in ruminants. **Methods:** Three recombinant *C. burnetii* antigens (Com-1, MceB, AdaA) were selected among immunodominant antigens and produced in a heterologous system (*Escherichia coli*). Following purification, the proteins were utilized to coat ELISA plates and evaluated for seroreactivity against sera from both negative and positive cattle. **Results:** Com-1 demonstrated the greatest agreement with the commercial test, albeit moderate. MceB exhibited nonspecific reactivity against a large number of sera, while the AdaA showed reactivity against only a few positive sera. **Conclusions:** Our findings are consistent with previous research, indicating that utilizing a single antigen to identify exposed animals is unfeasible with current knowledge, most likely due to the complex immunological response following *C. burnetii* infection in cattle. Consequently, it is critical to continue testing and identifying immunoreactive antigens in order to further investigate them and, potentially, select the most appropriate.

## 1. Introduction

*Coxiella burnetii* (*C. burnetii*) is the etiological agent of Q fever in humans, a globally reported public health concern, and Coxiellosis in ruminants, which causes economic losses in the cattle industry [1,2]. This intracellular and Gram-negative bacterium has a wide host range, including almost all mammals, and has been identified as one of the most common abortive agents in ruminants [1,3]. Coxiellosis in ruminants is often asymptomatic, with the sole symptoms being reproductive problems such as abortion, metritis, and placental retention [4,5]. Ruminants are considered the primary source of infection for humans, as they shed Coxiella in high concentrations through vaginal fluids and abortion products [6,7]. Other species, although susceptible to infection, appear to have a minor role in transmission [8,9,10]. Humans become infected through inhalation or ingestion of contaminated materials (some professionals, such as veterinarians and farmers, are at high risk) and may develop an acute form, characterized by fever and flu-like symptoms, or a chronic form, which is more dangerous due to Coxiella colonization in various organs such as the liver, lungs, and heart [1,11,12].

Ruminants are the main target for surveillance and monitoring programs due to their crucial role in the epidemiological cycle of Coxiella [11,13,14]. Surveillance strategies involve the detection of Coxiella DNA through direct methods, such as polymerase chain reaction testing on milk or aborted materials [15,16]. Additionally, serological tests are used to detect specific antibodies to assess the level of exposure within a herd [5,17]. Direct approaches are typically more expensive and are mainly recommended in outbreak situations, as intermittent shedding of Coxiella can impair diagnostic sensitivity [18,19]. Serology, on the other hand, is more suitable for rapid, large-scale screening. Previously, the complement fixation test (CFT) was the preferred serological tool, but it has largely been replaced by the immunofluorescence assay (IFA) and the enzyme-linked immunosorbent assay (ELISA) [18,20]. IFA is regarded as the gold standard for human diagnosis. It allows differentiation between recent and past exposure by detecting antibodies against two distinct antigenic phases of Coxiella: phase II (typical of recent and acute infection) and phase I (indicative of chronic infection) [1,21]. However, since no commercial IFA is currently available for veterinary use, ELISA is the most commonly used tool in ruminants and is also recommended by the World Organization for Animal Health (WOAH) [22]. These tests are relatively rapid, accessible, and easily applied to herd assessment, but they also have several drawbacks. In fact, commercial ELISAs are mostly based on whole-cell antigens of a single isolate, whose manufacturing requires a biosafety level 3 laboratory (BSL3) [23,24]. Furthermore, diagnostic performance may vary depending on the ruminant species or matrix used and may be influenced by antigenic homology between *C. burnetii* and other common ruminant pathogens, as well as the diversity of genotypes circulating in a given area [20,23,25,26].

Improving detection tools is essential for effective infection control, and numerous efforts have been made in recent years to develop recombinant ELISAs to address performance limitations and reduce the need for high-level biosafety procedures. Important immunodominant *C. burnetii* antigens (Ybgf, SucB, Hstp, etc.) have been employed in recombinant ELISAs. Comparative studies with commercial ELISAs have shown high concordance rates, although these alternatives have not been deemed suitable to fully replace existing commercial tests [24,27,28,29].

For these reasons, it is critical to continue identifying novel recombinant candidates, as the control of Q fever and Coxiellosis necessitates constant improvement of the assay used.

Several studies have proposed that major immunodominant proteins might be used to produce novel serological assays and vaccines, as they are often associated with a specific antigenic phase or form of infection [30,31]. These immunoreactive antigens include an outer membrane-associated protein (Com-1, CBU1910), acute disease antigen A (AdaA, CBU0952), and the mitochondrial Coxiella effector protein B (MceB, CBU0937) [32,33,34]. Com-1 is an outer membrane protein that participates in post-translational modification and protein turnover [35]. This antigen has been identified as immunodominant in multiple studies, demonstrating strong reactivity for both phases, and has already been used to detect anti-Coxiella antibodies in ruminants [36]. MceB and AdaA are two proteins that have demonstrated strong reactivity to phase II and are considered biomarkers of acute Coxiella infection [33].

The aim of this study was to produce these three antigens in a heterologous system (*Escherichia coli*), purify them, and employ them to coat ELISA plates in order to compare their diagnostic performance by testing positive and negative bovine sera.

## 2. Materials and Methods

### 2.1. Antigen Selection, Amplification, Ligation

A *C. burnetii* Nine Mile RSA 493 phase I strain was cultivated in axenic medium in a BSL3 laboratory in the department of Rickettiology, Slovak Academy Science, Bratislava (Slovakia) as described previously [37]. The bacteria were centrifuged at 15,000× *g* for 1 h at 4 °C, and genomic DNA was extracted using DNeasy blood and tissue kit (Qiagen, Venlo, The Netherlands) following the manufacturer’s instructions. Specific primers were designed according to the gene sequences available in the NCBI database (https://www.ncbi.nlm.nih.gov/; accessed on 1 October 2023), excluding the signal peptide region (Table 1). The primers included recognition sites for the restriction enzymes *BamHI* and *EcoRI* for cloning. However, since a restriction site for EcoRI was present in the sequence encoding for Com-1, this enzyme was replaced with XhoI (Supplementary Table S1). Each amplification was performed using a commercial kit (HotStarTaq DNA Polymerase, Qiagen) in a total volume of 50 μL including 2.5 μL of each primer (10 μM), 5 μL of buffer 10×, 1.5 μL of MgCl 2 (50 mM), 1 μL of dNTPs (10 mM), 0.25 μL of Taq DNA polymerase, and RNase-free water. PCR amplification was carried out under the following conditions: an initial denaturation at 95 °C for 5 min, followed by 35 cycles of denaturation at 95 °C for 45 s, annealing at 52–60 °C for 30 s, and elongation at 72 °C for 1–2 min depending on the amplicon length. A final elongation step at 72 °C for 10 min was also performed. Amplicons were visualized on agarose gel to confirm the presence and size of the expected bands. Digestion was performed at 37 °C for 3 h using appropriate restriction enzymes (BamHI, EcoRI, XhoI; Thermo Scientific, Waltham, MA, USA), followed by purification and quantification assessed using NanoDrop 2000/2000c spectrophotometer (Thermo Fisher, Waltham, MA, USA). Ligation was carried out as described in previous works [24] using specific plasmid vectors (pSER or pGEX 6P-1) previously digested with the same restriction enzymes. The ligation product was utilized for transforming competent *Escherichia coli* BL21 C43 (DE3) cells grown in Luria–Bertani (LB) media (Thermo Scientific, Waltham, MA, USA). Positive colonies were cultivated in ampicillin-supplemented liquid LB and stimulated with 1 mM isopropyl D-1-thiogalactopyranoside (IPTG) during the mid-exponential phase. The insert’s features and in-frame orientation were confirmed by colony PCR and by Sanger sequencing of the plasmid DNA extracted from a positive clone using the QIAprep Spin Miniprep kit (Qiagen, Venlo, The Netherlands).

### 2.2. Expression and Purification

Bacterial cells were collected by centrifugation (6000× *g* 10 min at 4 °C) and processed with 100 µL of lysozyme (50 mg/mL) in 10 mL of STE buffer (NaCl at 100 mM, Tris HCl at 10 mM, and Na2EDTA at 1 mM; Sigma-Aldrich, Burlington, MA, USA). Proteins were extracted and purified differently based on their solubility (presence or absence in the supernatant) using either denaturing or native conditions coupled to column-based chromatography. Specifically, MceB and AdaA (cloned into pSER vector) were purified using 1 M urea (from pellet) and a nickel-affinity resin (HisPur Ni-NTA resin; Thermo Scientific). Com-1, on the other hand, was cloned into the pGEX-6P-1 plasmid and purified from the supernatant using glutathione affinity chromatography (Glutathione Sepharose 4B resin; Merck, Rahway, NJ, USA), which also allowed the cut of the fusion GST tag from purified proteins directly on-column using PreScission protease (Merck, Rahway, NJ, USA) [24]. Each purification was carried out in three steps, after 30 min of resin incubation with protein extract and 5 min of incubation with a specific elution buffer, and visualized in Sodium Dodecyl Sulfate Polyacrylamide Gel Electrophoresis (SDS-PAGE) stained with Coomassie brilliant blue R250 (Sigma-Aldrich, Burlington, MA, USA).

### 2.3. Recombinant ELISAs

Each recombinant antigen was used (100 ng for Com-1; 50 ng for MceB and AdaA) to coat the wells of 96-well plates (Nunc Maxisorp; Millipore Sigma, Burlington, MA, USA) overnight at 4 °C, followed by a blocking phase with 2.5% bovine casein to prevent non-specific binding. Serum samples were diluted 1:20 for Com-1 and 1:100 for MceB/AdaA, and incubated for 1 h at room temperature. After three washes with a specific wash buffer, a monoclonal anti-bovine IgG-peroxidase was used as previously described [14]. After a final wash step, the enzymatic reaction was developed by adding 3,3′,5,5′-tetramethylbenzidine (TMB) for 15 min and then stopped with 0.2 M H_2_SO_4_ (Thermo Scientific, Waltham, MA, USA). Optical density was measured at 450 nm (OD450). A threshold for positivity was established as the mean OD of negative control sera plus four standard deviations (mean + 4SD). These operating conditions were determined by evaluating various antigen concentrations and dilutions of positive and negative control sera (crisscross serial dilution study with an initial panel of negative and positive serum samples) [14]. A panel of well-characterized sera belonging to apparently healthy ruminants and/or ruminants with a history of abortion was selected from a previous work [24] to investigate the diagnostic potential of recombinant antigens against a commercial ELISA (Q fever Ab test kit; IDEXX, Westbrook, ME, USA). The Ethics Committee of the Department of Veterinary Medicine and Animal Production (Centro Servizi Veterinari, Turin, Italy), University of Naples, Federico II, authorized the animal study protocol (PG/2022/0093419) on 20 July 2022. A total of 120 positive and 281 negative sera were selected from samples stored for other similar studies by choosing those best preserved and not thawed more than three times. The commercial ELISA was performed following the manufacturer’s instructions. Briefly, serum was diluted 1:100 to each well, incubated for 1 h at 37 °C, and, after three washes, 100 µL of anti-ruminant IgG conjugate was added. After further incubation and washing, the reaction was read by adding TMB for 15 min and stopping solution.

### 2.4. Statistical Analysis

The commercial ELISA (Q fever Ab test kit; IDEXX) was considered as the reference assay in our statistical analysis (MedCalc v.18.11.3, https://www.medcalc.org/; accessed on 1 December 2023). The level of agreement among assays was determined by the kappa Cohen coefficient (0.81–1.00 = almost perfect agreement; 0.61–0.80 = substantial agreement; 0.41 0.60 = moderate agreement; 0.21–0.40 = fair agreement; 0.01–0.20 = slight agreement; 0.00 = no agreement).

## 3. Results

**Figure 1:**
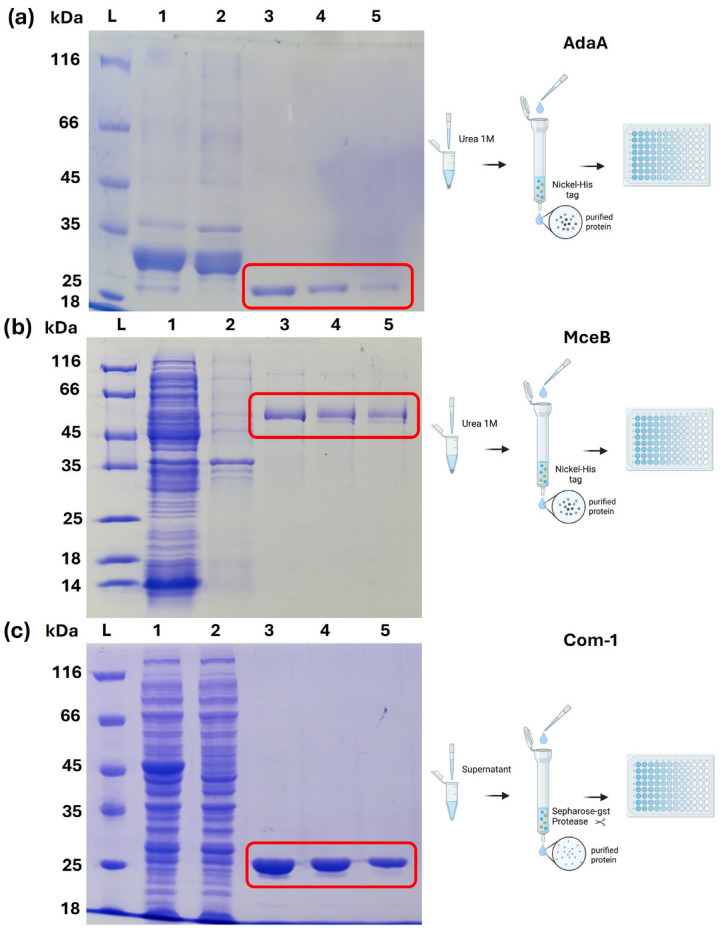
(**a**) SDS-PAGE analysis of the protein fractions obtained (AdaA): L = Ladder, 1 = 1 M urea extract, 2 = 1 M urea extract post-resin adsorption, 3, 4, 5 = fractions of the eluted protein. In well 1, a band can be observed corresponding to approximately 25 kDa. The intensity of this band is reduced in well 2 due to protein adsorption to the resin. In wells 3, 4, and 5, the protein purified under denaturing conditions is observed in three different eluates (indicated in red). (**b**) SDS-PAGE analysis of the protein fractions obtained (MceB): L = Ladder, 1 = 1 M urea extract, 2 = 1 M urea extract post-resin adsorption, 3, 4, 5 = fractions of the eluted protein. In well 1, a band can be observed corresponding to approximately 50 kDa. The intensity of this band is reduced in well 2 due to protein adsorption to the resin. In wells 4, 5, and 6, the protein purified under denaturing conditions is observed in three different eluates (indicated in red). (**c**) SDS-PAGE analysis of the fraction obtained (Com-1): L = Ladder, 1 = total extract, 2 = total extract after resin adsorption, 3, 4, and 5 = purified and cleaved protein eluates. Well 1 shows overexpression of a Com1 band around 50 kDa, corresponding to the fusion protein. Protein adsorption to resin reduces the intensity of this band in wells 2. Wells 3, 4, and 5 showed pure and cleaved proteins (indicated in red). After removing GST, the protein returns to its original molecular weight of 25 kDa. This figure has been created with the support of Biorender (Biorender.com, accessed on 1 May 2025).

All three recombinant antigens were successfully expressed and purified in sufficient quantities for use in ELISAs. Protein concentrations, as determined by the Bradford assay, were 1.2 mg/mL for Com-1, 0.4 mg/mL for MceB, and 0.55 mg/mL for AdaA. Purification of the target proteins for ELISA preparation was successful, as evidenced by SDS-PAGE electrophoresis (Figure 1a–c), which revealed a certain level of purity despite the presence of some additional protein residues in the final eluates. For Com-1, which was expressed as a GST fusion protein, proteolytic cleavage was performed directly on-column, allowing efficient removal of the fusion tag (Figure 1c). Among the three candidates, Com-1 showed the most promising diagnostic performance, demonstrating clear reactivity with both positive and negative serum samples, and was selected for further evaluation (and tested with a larger number of serum samples). In contrast, the concordance for AdaA was poor (overall agreement of 0.54) as the antigen only reacted with 17/55 positive sera (Table 1 and Table 2). Similarly, MceB demonstrated a low concordance (0.56), largely due to a high number of false positives (37/60) (Table 1 and Table 2). In both cases, Cohen’s kappa values ranged between 0 and 0.2, revealing poor agreement with the commercial test. Com-1 achieved an overall agreement of 0.83 with a Cohen’s kappa value of 0.58 which was considered a moderate agreement. Furthermore, the Com-1-based ELISA appeared to be more specific than sensitive, as fewer false positives (28/281) than false negatives (40/120) were observed.

## 4. Discussion

Three immunodominant antigens of *C. burnetii* were produced in recombinant form in this work and were evaluated for their ability to detect specific antibodies in bovine sera. Among the candidates, Com-1 was the only antigen that yielded promising results, correctly identifying 80 out of 120 positive animals and 253 out of 281 negative animals. This antigen reached a moderate agreement (0.83) when it was compared to a commercial ELISA kit. For this reason, it was evaluated with a greater number of serum samples.

These results are consistent with a previous study in which Com-1 was produced in recombinant form, albeit with different production and purification methods. Reported diagnostic sensitivity and specificity ranged between 65 and 85% depending on the species, with better diagnostic performance in goats than in cattle [38]. A further study described the increase in diagnostic performance for the Com-1 antigen (around 83% sensitivity and 80% specificity) when it was produced synthetically and utilized in the latex agglutination test (LAT) [39].

Studies focusing on other antigens have found similar results, regardless of the test format or ruminant species. Although reported to be immunodominant in both humans and animals, antigens such as cell division coordinator CpoB (Ybgf) or dihydrolipoyllysine-residue succinyltransferase (SucB) have achieved high specificities (around 90%) but lower sensitivity (70–80%) in various ELISA formats [24,27,40]. The reported concordance values were 0.83 for SucB and 0.86 and 0.88 for Ybgf when used in double-antigen ELISA or indirect ELISA, respectively [24,41]. Currently, the most effective recombinant antigen described was heat shock protein B (HspB) with sensitivity around 80–90% and high specificity (97%) [28]. However, this antigen has only been tested in experimentally infected goats [28].

All these findings should be interpreted in the context of the complex immune response elicited by Coxiella infection. As in humans, ruminants typically generate antibodies to phase II antigens during acute infection and seroconvert to phase I antigens as the infection becomes chronic [42]. For this reason, commercial kits consist of antigen mixtures from both phases to maximize detection, although this feature can reduce specificity due to potential cross-reactivity with other pathogens. Conversely, single recombinant antigens, while potentially more specific, may lack sensitivity if they only represent one phase or stage of the immune response [27]. However, it is noteworthy that the performance of the commercial test utilized as a reference in this study was not without limitations. In the absence of a true gold standard or validated reference sera, the reliability of commercial tests has been questioned, in particular considering the high rates of false-positive and false-negative results reported in several studies [43,44,45]. This is a common issue in the evaluation of recombinant antigens for the detection of anti-Coxiella antibodies in veterinary medicine. The WOAH manual recommends ELISA as the reference test for serology, despite its limitations associated with false positives caused by cross-reactions with organisms that share Coxiella’s antigenic profile (such as Bartonella, Rickettsiae, etc.) [14].

Another explanation for the low concordance of recombinant antigens could be associated with the inability of *Escherichia coli* (used as the expression system) to perform post-translational modifications, such as glycosylation or methylation, that can be important for the correct epitope conformation and immune recognition [24].

Furthermore, the genetic diversity of *C. burnetii* strains and their association with different ruminant species and the enzootic areas may affect reactivity. For instance, more virulent strains possess the AdaA gene, while others lack it. Additionally, the AdaA gene has been identified predominantly in sheep, which could explain its low diagnostic performance in cattle sera [30].

While none of the tested antigens are yet suitable for immediate use in routine screening, Com-1 stands out as a promising candidate for further research and development. Further studies should evaluate this antigen using a larger number of well-characterized sera, including samples from experimentally infected/vaccinated animals or reference sera.

Com1 is an outer membrane protein that displays reactivity for both phases, although its specificity may be compromised by cross-reactions with some rickettsial species [34,46]. It is described in the literature as being more reactive in chronic forms, and to date, more than 15 articles have highlighted its immunoreactive properties, identifying it as a major immunodominant antigen in humans and a strong candidate for future applications in serology and vaccinology [33,36]. One human investigation showed Com-1 reacted with sera from individuals with chronic Q fever, agreeing with 92.4% (122/132) of negative clinical diagnoses and 72.2% (26/36) of positive clinical diagnoses [47]. However, veterinary research on Com-1 remains limited, and its full potential in animal diagnostics has yet to be fully explored. Improving ruminant screening tests is a veterinary priority for Q fever prevention. It is a significant concern that, despite advances in human serology (with IFA and chemiluminescent assays), which allows for fine-tuned diagnosis, differentiation of vaccinated from infected patients, determination of the infection stage, and assessment of treatment efficacy, veterinary serology still lacks reliable and standardized tools [22]. More attempts are clearly necessary, both to develop new assays and to enhance existing ones.

## 5. Conclusions

In this work, the potential of three recombinant antigens to detect specific anti-Coxiella antibodies in ELISA was tested using cattle serum samples. Com-1 showed the best agreement with a commercial ELISA kit and demonstrated potential as a candidate for further investigation. Although the data do not support the use of this Com-1-based ELISA as a screening test in ruminants at this time, they justify additional research into its applicability for serodiagnosis in veterinary medicine.

## Figures and Tables

**Table 1:** Positive and negative cattle serum samples tested using a commercial ELISA (IDEXX) and the three recombinant ELISAs (MceB, AdaA and Com-1).

Species	IDEXX+	IDEXX−	Total
MceB+	41	37	
MceB−	14	23	
Total	55	60	115
AdaA+	17	15	
AdaA−	38	45	
Total	55	60	115
Com-1+	80	28	
Com1−	40	253	
Total	120	281	401

Abbreviations: + = positive; − = negative.

**Table 2:** Comparison of performance of recombinant ELISAs (MceB, AdaA and Com-1) and a commercial ELISA (IDEXX) using cattle serum samples.

Species	Agreement (95% CI)	Cohen κ (95% CI)
MceB	0. 56 (46.1–64.9)	0.13 (0–0.29)
AdaA	0.54 (44.4–63.2)	0.06 (0–0.22)
Com-1	0.83 (79–86.6)	0.58 (0.49–0.67)

CI = Confidence Intervals.

## Data Availability

The original contributions presented in this study are included in the article/Supplementary Material. Further inquiries can be directed to the corresponding author.

## Supplementary Materials

The following supporting information can be downloaded at: https://www.mdpi.com/article/10.3390/antib14040107/s1, Table S1: Primers used.

## Abbreviations

The following abbreviations are used in this manuscript:

CFT	Complement fixation test
ELISA	Enzyme-linked immunosorbent assay
IFA	Immunofluorescence Assay
LAT	Latex agglutination test
GST	Glutathione S-Transferase
TMB	3,3′,5,5′-tetramethylbenzidine
IPTG	Isopropyl β-d-1-thiogalactopyranoside
WOAH	World Organisation for Animal Health
