# Supplementary material for: Evaluation of Three Recombinant Antigens for the Detection of Anti-Coxiella Antibodies in Cattle
Source: Antibodies (Basel). 2025 Dec 12;14(4):107. doi: 10.3390/antib14040107 (PMC12729987; doi:10.3390/antib14040107)
Supplement: Supplementary file 1 [file antibodies-14-00107-s001.zip › antibodies-3957609-supplementary.pdf]

Table S1: Primers used

Protein/Gene	Forward (5'-3')	Reverse (5'-3')	Size (bp)	Plamid used
Com-1 (CBU_1910)	TTGGATCCCAAGTCAAAGACATACAAAGCATCG	TTCTCGAGCTTTTCTACCCGGTCGATTTC	660	pGEX-6P
AdaA (CBU_0592)	TTGGATCCGAAAATCGCCCCATTTTAAATACG	TTGAATTCTGTTTTTTCAGACGGGGCAGATC	545	pSer
MceB (CBU_0937)	TTGGATCCCCCGCTACCACTAATCAACAAATCAC	TTGAATTCATAAAGATCGAACTGTGCCGTTACCAC	1,300	pSer
